# Impact of Global *Fxr* Deficiency on Experimental Acute Pancreatitis and Genetic Variation in the *FXR* Locus in Human Acute Pancreatitis

**DOI:** 10.1371/journal.pone.0114393

**Published:** 2014-12-03

**Authors:** Rian M. Nijmeijer, Frank G. Schaap, Alexander J. J. Smits, Andreas E. Kremer, Louis M. A. Akkermans, Alfons B. A. Kroese, Ger. T. Rijkers, Marguerite E. I. Schipper, André Verheem, Cisca Wijmenga, Hein G. Gooszen, Karel J. van Erpecum

**Affiliations:** 1 Department of Surgery, University Medical Center Utrecht, Utrecht, the Netherlands; 2 Tytgat Institute for Liver and Intestinal Research, Academic Medical Center, Amsterdam, the Netherlands; 3 Department of General Surgery, Maastricht University, Maastricht, The Netherlands; 4 Department of Pathology, University Medical Center Utrecht, Utrecht, the Netherlands; 5 Department of Medicine 1, Friedrich-Alexander-University of Erlangen-Nuremberg, Erlangen, Germany; 6 Neurotoxicology Research Group, Institute for Risk Assessment Sciences, Utrecht University, Utrecht, the Netherlands; 7 Department of Operating Rooms, University Medical Center St. Radboud, Nijmegen, the Netherlands; 8 Department of Medical Microbiology and Immunology, St. Antonius Hospital, Nieuwegein, the Netherlands; 9 Department of Genetics, University Medical Centrum Groningen, University of Groningen, Groningen, the Netherlands; 10 Department of Gastroenterology and Hepatology, University Medical Center Utrecht, Utrecht, the Netherlands; IRCCS Istituto Oncologico Giovanni Paolo II, Italy

## Abstract

**Background:**

Infectious complications often occur in acute pancreatitis, related to impaired intestinal barrier function, with prolonged disease course and even mortality as a result. The bile salt nuclear receptor farnesoid X receptor (FXR), which is expressed in the ileum, liver and other organs including the pancreas, exhibits anti-inflammatory effects by inhibiting NF-κB activation and is implicated in maintaining intestinal barrier integrity and preventing bacterial overgrowth and translocation. Here we explore, with the aid of complementary animal and human experiments, the potential role of FXR in acute pancreatitis.

**Methods:**

Experimental acute pancreatitis was induced using the CCK-analogue cerulein in wild-type and *Fxr^-/-^* mice. Severity of acute pancreatitis was assessed using histology and a semi-quantitative scoring system. Ileal permeability was analyzed *in vitro* by Ussing chambers and an *in vivo* permeability assay. Gene expression of *Fxr* and *Fxr* target genes was studied by quantitative RT-PCR. Serum FGF19 levels were determined by ELISA in acute pancreatitis patients and healthy volunteers. A genetic association study in 387 acute pancreatitis patients and 853 controls was performed using 9 tagging single nucleotide polymorphisms (SNPs) covering the complete *FXR* gene and two additional functional SNPs.

**Results:**

In wild-type mice with acute pancreatitis, ileal transepithelial resistance was reduced and ileal mRNA expression of *Fxr* target genes *Fgf15*, *SHP*, and *IBABP* was decreased. Nevertheless, *Fxr^-/-^* mice did not exhibit a more severe acute pancreatitis than wild-type mice. In patients with acute pancreatitis, FGF19 levels were lower than in controls. However, there were no associations of *FXR* SNPs or haplotypes with susceptibility to acute pancreatitis, or its course, outcome or etiology.

**Conclusion:**

We found no evidence for a major role of FXR in acute human or murine pancreatitis. The observed altered Fxr activity during the course of disease may be a secondary phenomenon.

## Introduction

Acute pancreatitis (AP) is the acute inflammation of the pancreas, and is mostly caused by gallstones or alcohol abuse [1]. In the majority of patients the course of the disease is mild, but in around 20% of patients, AP is severe with organ failure and/or local complications [2]. Mortality from AP is especially caused by infectious complications, such as bacterial infection of pancreatic necrosis [3], [4]. Failure of the intestinal barrier function plays a critical role, as it allows for bacterial translocation, facilitating such infectious complications [5]-[8].

The intracellular bile salt receptor farnesoid X receptor (FXR) is mainly expressed in ileum and liver, and to some extent in other organs, such as the pancreas [9], with little information available on its function in the latter organ. FXR is considered the master regulator of bile acid homeostasis, which regulates various genes encoding for bile acid transport proteins, including apical sodium-dependent bile acid transporter (ASBT) and ileal bile acid binding protein (IBABP) [10], [11]. Also, the enterokine fibroblast growth factor 15 (Fgf15, human orthologue FGF19), whose expression is controlled by FXR, exerts a negative feedback regulation of hepatic bile salt neo-synthesis and, at least in mice, induces gallbladder refilling at the end of the postprandial phase [12].

More recently, FXR has been implicated in the regulation of fat and glucose metabolism, in the maintenance of intestinal barrier integrity and prevention of intestinal bacterial overgrowth, by affecting putative FXR-dependent genes such as angiogenin-1, iNOS, CAR12 and IL18 [13]. In patients with Crohn's colitis, who show impaired antibacterial defense and impaired intestinal barrier function, FXR expression was altered in areas of inflamed mucosa [14]. Furthermore, we recently showed in two murine models for colitis that administering the semi-synthetic FXR agonist INT747 (Obeticholic acid) ameliorates intestinal inflammation, improving colitis symptoms, preserving intestinal barrier function, and reducing goblet cell loss [15]. The underlying mechanism for these anti-inflammatory effects is thought to be inhibition of NF-κB [16]. We also recently detected impaired mRNA expression of FXR target genes in the ileum of patients with clinically quiescent Crohn's colitis [17]. FGF19 signaling has been implicated in regulating inflammation by antagonizing NF-κB signaling in FGF19 target tissues, which may include the pancreas [18], [19].

Because of its role in intestinal barrier function, *i.e.* prevention of bacterial translocation and modulation of inflammation, we hypothesized that FXR might play an important role in AP. Deficiency of FXR could result in increased severity of the pancreatitis, increased bacterial translocation, and infectious complications. In this study, we therefore explored, with the aid of complementary animal and human experiments, whether FXR could affect AP.

## Materials and Methods

### Animals

In the first series of experiments, we used adult male wild-type C57BL/6 mice of 10–12 weeks and 20–30 grams of weight (Harlan, Horst, the Netherlands). For the second series of experiments, mice with global *Fxr* deficiency (*Fxr^-/-^*) on a C57BL/6 genetic background [20] were obtained by breeding of heterozygous mice. We used male adult *Fxr^-/-^* and wild-type C57BL/6 littermates of 11–16 weeks and 25–35 grams of weight. All mice were kept under constant housing conditions (22°C, 60% relative humidity and a 12-hour light/dark cycle) for at least two weeks prior to the start of the experiment, and had free access to water and food (CRM (E), B.M.I. – Technilab, Someren, the Netherlands) throughout the experiment.

### Animal experiments

AP was induced by ten intraperitoneal injections with an hourly interval of cerulein, a CCK analogue (Sigma-Aldrich Chemie B.V., Zwijndrecht, the Netherlands; 50 µg/kg in 0.9% NaCl). Controls received an equal volume of saline. In an initial experiment, pancreatic injury was assessed 24 and 72 hrs after induction of AP. For this purpose, 30 wild-type mice (Harlan) were randomly allocated to a control group (n = 10, sacrificed after 72 hrs) and two experimental groups that were terminated after 24 hrs (early pancreatitis, n = 10) and 72 hrs (late pancreatitis, n = 10). To assess the impact of *Fxr* deficiency on AP, *Fxr^+/+^* (wild-type) and *Fxr^-/-^* mice received control (n = 5) or cerulein (n = 10 per genotype) treatment and were sacrificed after 24 hrs.

Animals were terminated by cervical dislocation or by cardiac puncture under isoflurane anesthesia. For histopathologic evaluation, parts of the ileum and pancreas were fixated in 4% formaldehyde. For RNA isolation, parts of the ileum and liver were immediately snap frozen in liquid nitrogen and stored at −80°C. Plasma samples were stored at −80°C for determination of amylase and bilirubin by standard clinical chemical assays.

The experimental design was approved by the animal experiments committee of Utrecht University, Utrecht, the Netherlands (2007.III.09.117; 2009.III.08.074).

### Histopathology

After fixation in 4% formaldehyde, tissues were embedded in paraffin and cut in serial sections of 4 µm for hematoxylin and eosin (H&E) staining. Qualitative assessment of the severity of AP was performed in the initial experiment and, in the second experiment, a slightly modified semi-quantitative scoring system was used [21], [22]. The following items were scored: edema (0-4 points), number of neutrophils in the edema (0–4 points), pancreatic ductal pathology (inflammatory cells; present = 1; absent = 0), intralobular inflammatory infiltrate (0–3 points) and peripheral necrosis of pancreatic tissue (0–4 points). The maximum composite score was 16. To assess the ileal brush border in the second experiment, PAS-diastase staining was performed. Histopathological evaluation was performed by two experienced pathologists (AJJS, MEIS), blinded for experimental study groups.

### Measurement of transepithelial electrical resistance

In the initial experiment, a 4 cm segment of the distal ileum was removed for electrical resistance measurements in Ussing chambers, as described elsewhere [22]. Briefly, flat sheets of mucosa were mounted in Ussing chambers with both sides of the epithelium in contact with Krebs-Ringer's solution, stirred and gassed with humidified carbogen at 37°C. Three ileal samples per animal were used. The transepithelial potential difference V_te_ (mV) was continuously monitored and transepithelial electrical resistance R (Ω.cm^2^) was calculated [22]. The reported values for the resistance were obtained at the end of the 20 min equilibration period. At the end of the experiment, viability of the tissue segments was confirmed based on carbachol-induced voltage increase [22].

### In vivo intestinal permeability assay

In the second experiment, intestinal permeability was assessed with fluorescein isothiocyanate (FITC)-conjugated dextran as previously described [15]. Briefly, two hours before termination, mice were gavaged with 0.6 mg/g body weight of FITC-conjugated dextran (MW 3,000–5,000 Da; Sigma-Aldrich). After termination, FITC fluorescence was measured in plasma with the aid of a fluorometer (BMG Polarstar Galaxy, MTX Lab Systems, Inc., Vienna, Virginia, USA) and compared to a calibration line of standard concentrations of FITC-conjugated dextran.

### Analysis of gene expression

Total RNA was isolated from murine ileum and liver (RNeasy Midi Kit, Qiagen, Hilden, Germany). RNA integrity was tested by RNA gel electrophoresis. cDNA was synthesized from total RNA using the iScript cDNA synthesis kit (BioRad, Hercules, CA, USA). Quantitative RT-PCR was performed using SYBR Green Supermix (BioRad) on an iCycler iQ system using diluted cDNA as template (primer sequences are provided in Table S1 in File S1). Expression levels were estimated using the comparative threshold cycle method. Cyclophilin was used as housekeeping gene, with similar expression levels in ileum and liver under all experimental conditions.

### Determination of plasma FGF19 levels in patients with acute pancreatitis

FGF19 levels were determined by ELISA in plasma samples of 15 randomly selected patients with predicted severe AP [23]. Patients were participants in an earlier clinical trial (trial registry ISRCTN38327949) and were fed by continuous enteral nutrition [24]. Clinical data were available from the prospectively collected trial database [24]. As a control group, FGF19 levels were also determined in a group of 28 healthy volunteers receiving an oral fat load [25]. In this group, fed FGF19 levels were calculated as the average of postprandrial FGF19 levels at 2, 3, 4, and 6 hrs.

### Genetic association study

For the genetic association study, a previously described cohort of 387 patients with a first episode of AP was used [26]. All patients or their legal representatives gave their written informed consent, and the ethics review boards of all participating hospitals approved the study protocol. Genomic DNA was isolated from whole blood using a DNA isolation kit I (Magna Pure LC, Roche Diagnostics, Indianapolis, USA). Clinical data on the severity of disease and outcome of all patients were available from the prospectively collected trial database [24]. The controls consisted of 853 healthy, voluntary, Dutch blood donors [27]. All control genotypes were in Hardy-Weinberg equilibrium (data not shown, p>0.05). Call rates for all SNPs were>95%.

Nine tagging single nucleotide polymorphisms (SNPs) covering the complete *FXR* gene were selected using Haploview v4.2 [28]. In addition, two functional SNPs affecting FXR expression (-1G/T, rs56163822) and FXR function (518T/C, rs61755050) were analyzed [29]. Details of the SNPs studied are given in Table S2 in File S1. Genotyping was performed using TaqMan assays on a TaqMan 7900 HT (Applied Biosystems, Foster City, California, USA). Haplotype analysis was performed in Haploview [28].

### Statistical analysis

Statistical analyses were performed using GraphPad PRISM software (Graphpad Software, La Jolla, CA, USA). Electrical resistance, histology scores, and clinical parameters were compared using one-way ANOVA with Tukey's post-hoc test or the non-parametric Kruskal-Wallis test with Dunn's post-hoc test where appropriate. Differences in gene expression levels were evaluated using the non-parametric Kruskal-Wallis test with Dunn's post-hoc test. Plasma FGF19 levels were compared between AP patients and healthy controls by ANOVA with Tukey's post-hoc test. Statistical analysis of the genetic association study was performed using two-tailed chi-squared for independence tests of case versus control allele and haplotype counts in Haploview v4.2 [28]. Uncorrected P-values, odds ratios (OR) and 95% confidence intervals (95% CI) are given (Table 1 and Table S3 in File S1). The Bonferroni method was used to correct for multiple testing. Data of continuous values are shown as mean ± standard deviation (SD). P-values below 0.05 were considered statistically significant.

**Table 1:** Association of genetic variants of *FXR* with acute pancreatitis.

		Acute pancreatitis patients			Controls			P value*	OR	95% CI
		Allele counts			Allele counts					
		Major	Minor	MAF	Major	Minor	MAF			
-1 G>T	C/A#	732	14	0.981	1588	36	0.978	0.5926	1.14	0.62–2.10
518 T>C	A/G	743	5	0.993	1616	6	0.996	0.3203	1.86	0.60–5.82
rs11837065	C/T	452	250	0.644	1014	592	0.631	0.5663	1.05	0.88–1.27
rs12313471	A/G	699	39	0.947	1548	76	0.953	0.5268	1.15	0.77–1.71
rs11110390	C/T	497	247	0.668	1070	544	0.663	0.8088	1.02	0.85–1.23
rs4764980	G/A	384	354	0.520	832	778	0.517	0.8728	1.01	0.85–1.21
rs11110395	G/T	692	44	0.940	1538	84	0.948	0.4272	1.18	0.81–1.71
rs17030285	C/G	610	102	0.857	1415	215	0.868	0.4599	1.11	0.86–1.42
rs11610264	T/C	536	200	0.728	1160	458	0.717	0.5703	1.06	0.87–1.28
rs10860603	G/A	616	122	0.835	1398	214	0.867	0.0364	1.30	1.02–1.65
rs35739	T/C	398	350	0.532	900	712	0.558	0.2334	1.11	0.93–1.32

OR  =  odds ratio; 95% CI  = 95% confidence interval.

# Major allele/minor allele; MAF  =  major allele frequency.

*Two-tailed p values were calculated by χ^2^ analysis of allele count.

## Results

### Acute pancreatitis results in decreased transepithelial resistance and altered expression of Fxr targets in the ileum

**Figure 1:**
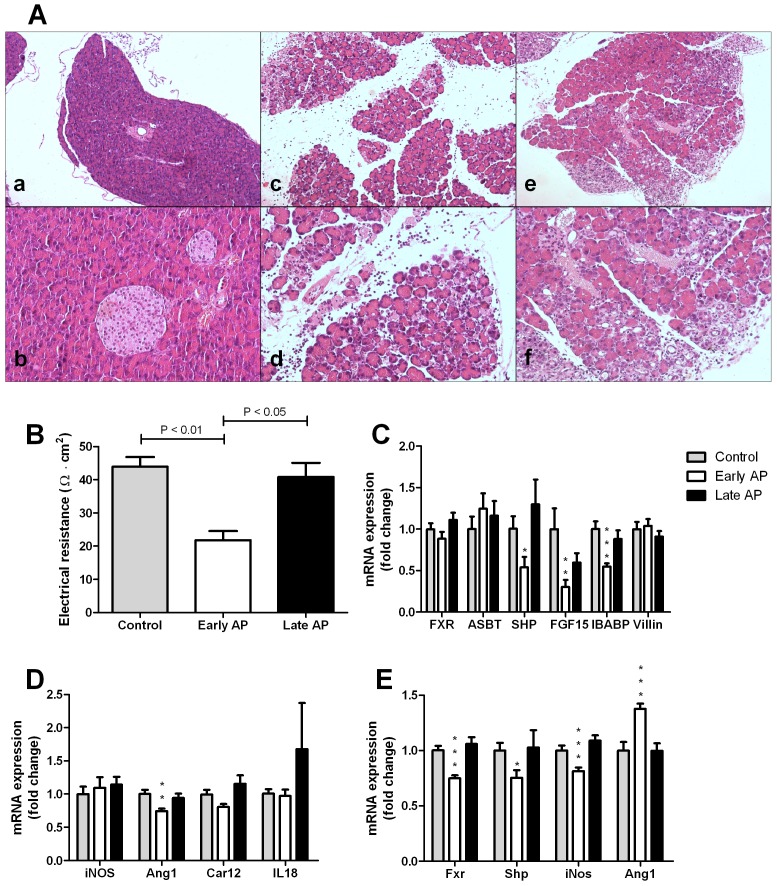
A – Representative pancreatic histology of wild-type mice from the control group (a, b) and mice with early (c, d) and late (e, f) acute pancreatitis (H&E staining, 20x and 100x magnifications consecutively). Control mice have normal pancreatic morphology, whereas mice of the early pancreatitis group exhibit edema, influx of neutrophils and necrosis. Mice of the late pancreatitis group have no edema or necrosis, but show influx of lymphocytes and fibroblasts. B – Transepithelial electrical resistance of the ileum measured by Ussing chamber experiments. The resistance of the ileum was lower in the early pancreatitis group in comparison to both controls and the late pancreatitis group. C – Ileal mRNA expression of *Fxr* and FXR target-genes *Asbt*, *Shp*, *Fgf15*, and *Ibabp*, and *Villin* in wild-type mice of the control group, and the early and late pancreatitis groups. Expression of *Fxr*, *Asbt* and *Villin* did not differ between experimental groups. Expression of the other Fxr target genes was lower in early acute pancreatitis, but not in late pancreatitis. D – Ileal mRNA expression of genes implicated in intestinal barrier function, *iNos*, *Ang1*, *Car12*, *IL18*. Expression of *Ang1* was lowered in the early pancreatitis group, the other genes remained similar in the different experimental conditions. E – Hepatic expression of *Fxr*, *Shp*, *iNos* and *Ang1*. Hepatic *Ang1* was increased in early pancreatitis, whereas the expression of the other genes was lowered in the early acute pancreatitis group. Expression levels were normalized to cyclophilin expression. Bars indicate means and SEM, * p<0.05, ** p<0.01, *** p<0.001.

Pancreatic injury was initially assessed in wild-type mice sacrificed at 24 hrs (early AP) or 72 hrs (late AP) after induction of AP. Plasma amylase levels were twice as high in the early and late AP mice compared to the control group (mean ± SEM, 4521±527 U/L vs. 2186±109 U/L, p<0.001). Histopathological examination of the pancreas revealed edema, influx of neutrophils and necrosis in all mice of the early pancreatitis group (Figure 1A). In contrast, the pancreata of all mice in the late pancreatitis group showed no signs of edema or necrosis and displayed infiltration of lymphocytes and fibroblasts rather than neutrophils. There were no histopathological abnormalities in the control group. Histopathological examination of the ileum revealed normal enterocytes without any signs of inflammatory infiltrate in all groups. Nevertheless, the transepithelial electrical resistance of the ileum was approximately half in the early AP group compared to controls and the late AP group (Figure 1B). This indicates that AP induces a transient increase in ileal permeability.

Impaired intestinal barrier function in patients with inflammatory bowel disease is accompanied by reduced ileal expression of FXR targets [17]. Ileal gene expression in mice was therefore analyzed to test the consequences of an AP-induced decline of transepithelial electrical resistance. *Fxr* mRNA expression was comparable between the three experimental groups, as was mRNA expression of the Fxr-target gene *Asbt* (Figure 1C). In contrast, mRNA expression of Fxr-target genes *Shp*, *Fgf15* and *Ibabp* was reduced in the early AP group compared to control mice (Figure 1C). In the late pancreatitis group, expression of all Fxr target genes was normalized (Figure 1C). *Fxr* and its target genes are exclusively expressed in the villous lining of differentiated enterocytes [13]. We therefore also assessed mRNA expression of *Villin*, which is expressed exclusively in these differentiated enterocytes [17]. mRNA expression of *Villin* showed no differences between the groups, including the early AP group (Figure 1C), indicating that no intestinal damage was present in this mouse model of AP. Regarding Fxr-dependent genes implicated in intestinal barrier function [13]: *Angiogenin-1* (*Ang1*) mRNA expression in the ileum was reduced in the early pancreatitis group, whereas *iNos*, *Car12* and *Il18* were similar in all groups (Figure 1D).

In the liver, mRNA expression of *Fxr* and its target gene *Shp* were diminished after 24 hours, but normalized after 72 hours (Figure 1E). *Fgf15* could not be detected in the liver. Hepatic *iNos* expression was also lowered after 24 hours, whereas *Ang1* expression increased in the early phase and returned to baseline expression in the late phase (Figure 1E).

### Deficiency of Fxr does not lead to more severe acute pancreatitis in mice

The above findings indicate that ileal Fxr activity is disturbed in the early phase of murine AP (i.e. 24 hrs after induction). To test whether Fxr dysfunction contributes to the pathology of AP, mice deficient for Fxr were given ten hourly injections of cerulein to induce AP and sacrificed after 24 hrs. Weight loss due to pancreatitis induction did not differ between wild-type and Fxr^-/-^ mice (mean ± SEM: 6.1±0.37 and 5.2±1.78% of body weight, respectively, p = 0.48). In both wild-type and Fxr^-/-^ pancreatitis groups, plasma amylase levels were significantly higher than in corresponding groups without AP (mean ± SEM; wild-type controls 2160±149 U/L, wild-type AP 8013±923 U/L, p<0.01; Fxr^-/-^ controls without AP 2285±96 U/L, Fxr^-/-^ AP 6801±671 U/L, p<0.01). C-reactive protein (CRP) in serum of mice of all four experimental groups was always very low (<5 mg/L), indicating absence of significant systemic inflammation.

In order to identify whether cholestasis was present in these mice as a sign of post-hepatic bile duct obstruction by the inflamed pancreas, we determined plasma bilirubin levels. In wild-type and Fxr^-/-^ mice, AP did not affect the plasma bilirubin levels (mean ± SEM; wild-type controls 1.8±0.5 µmol/L, wild-type AP 1.5±0.2 µmol/L; Fxr^-/-^ without AP 8.0±2.1 µmol/L, Fxr^-/-^ with AP 5.8±2.6 µmol/L). Fxr deficiency resulted in elevated bilirubin levels (p<0.05). As a potential explanation for this phenomenon, we found elevated hepatic expression of the basolateral bilirubin glucuronide efflux pump Mrp1 in Fxr^-/-^ mice (data not shown) [30].

We subsequently investigated whether Fxr deficiency affects epithelial permeability. Plasma levels of FITC-conjugated dextran were not increased by AP induction in either wild-type or Fxr^-/-^ mice. Nevertheless, Fxr^-/-^ mice had significantly higher plasma levels of FITC-conjugated dextran than wild-type mice (mean ± SEM: wild-type, 3.41±0.61 µg/ml vs. Fxr^-/-^, 7.45±2.31 µg/ml, p<0.05), indicating that loss of Fxr leads to increased intestinal permeability.

**Figure 2:**
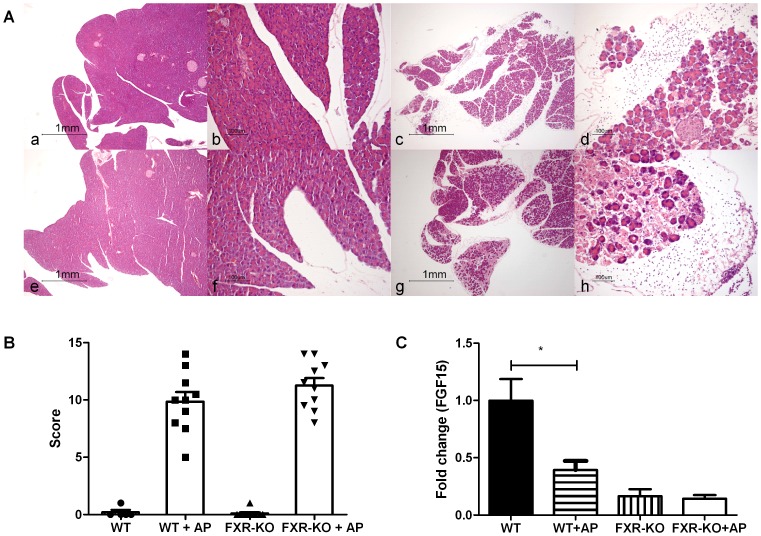
A - Representative pancreatic histology following induction of acute pancreatitis (H&E staining, 20x and 100x consecutive magnifications for each experimental group): wild-type control (a,b); wild-type acute pancreatitis (c,d); Fxr^-/-^ control (e,f); Fxr^-/-^ acute pancreatitis (g,h). B – Semi-quantitative composite pancreatitis severity score of histopathological examination of pancreas samples from wild-type and Fxr^-/-^ mice with and without acute pancreatitis. Absence of *Fxr* does not result in more severe acute pancreatitis. C – Ileal mRNA expression of *Fgf15* in wild-type and Fxr^-/-^ mice with and without early acute pancreatitis. *Fgf15* expression was decreased in wild-type mice with acute pancreatitis. Bars indicate mean and SEM.

Upon histopathological examination, wild-type and Fxr^-/-^ control mice did not exhibit edema, influx of inflammatory cells, or necrosis of the pancreas. In contrast, all mice in the pancreatitis groups showed clear signs of AP: interlobular and/or interacinar edema, influx of neutrophils, and necrosis (Figure 2A). Pancreatitis severity scores were similar in wild-type and Fxr^-/-^ mice (composite pancreatitis severity score, mean ± SEM: 9.9±0.8 vs. 11.3±0.7, p = 0.27; Figure 2B). When the individual components of the severity score (presence of edema, inflammatory infiltrate, and necrosis), were analyzed, there were also no differences found between wild-type and Fxr^-/-^ mice (data not shown).

As expected, AP did not affect the expression of *Fxr* in the ileum of wild-type mice (results not shown). In contrast to the results depicted in Figure 1C, the effects of AP on expression of Fxr targets *Shp* and *Ibabp* in the ileum of wild-type mice did not reach significance in this experiment (results not shown). Nevertheless, a consistent decrease in expression of Fxr target *Fgf15* in the ileum was still noted following induction of AP (Figure 2C). Deficiency of Fxr resulted in reduced ileal expression of Fxr targets *Shp* (data not shown) and *Fgf15* (Figure 2C) in mock-treated mice, with AP having no additional suppressive effect. Fxr mRNA could still be detected, albeit at a lower level, in the ileum of Fxr^-/-^ mice. The strategy used for disruption of the Fxr gene in these mice [20] results in a non-functional transcript as is evident from the near absence of ileal Ibabp expression in Fxr^-/-^ mice (results not shown).

Upon histopathological examination, there were no inflammatory infiltrates in the ileum of wild-type or Fxr^-/-^ mice with and without AP and PAS-diastase staining showed intact brush borders (results not shown). There were no differences in mRNA expression of inflammatory genes *Car12* and *iNos* in the enterocyte (results not shown). As an additional marker of pro-inflammatory response, we determined *Tnf-α* mRNA expression, but found no differences in expression (results not shown). These findings indicate that there were no signs or very limited signs of inflammation on the molecular level in the ileum.

### Plasma FGF19 levels are lowered in patients with acute pancreatitis

**Figure 3:**
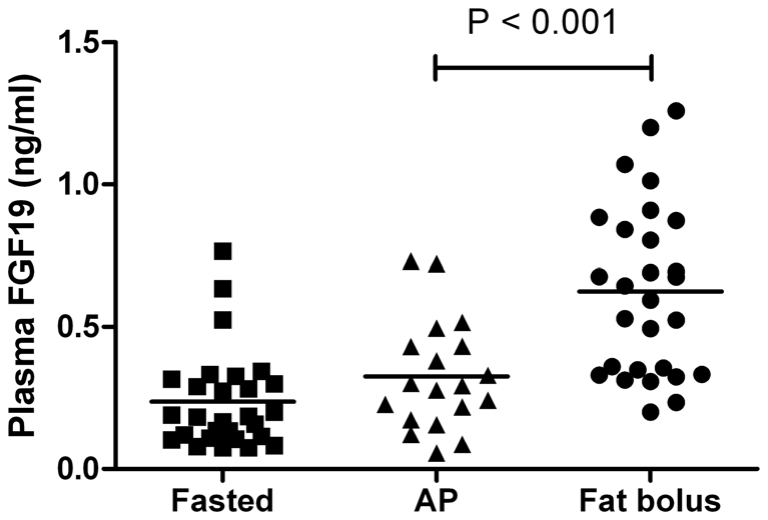
FGF19 plasma levels in patients with predicted severe acute pancreatitis during continuous enteral nutrition. For comparison, FGF19 plasma levels of healthy controls in the fasting state and after a single bolus of fat are also shown. The postprandial levels represent the average of plasma FGF19 levels at 2, 3, 4 and 6 hours after fat ingestion. There appears to be a blunted FGF19 release in the pancreatitis group. Bars indicate mean and SD.

To obtain an impression of FXR activation in patients with AP, we studied plasma FGF19 levels in patients with predicted severe pancreatitis. Plasma FGF19 levels in AP patients under digestive conditions were significantly lower than in healthy volunteers after ingestion of a single bolus of fat (0.33±0.19 vs. 0.62±0.30 ng/mL, p<0.001; Figure 3). This suggests that in patients with AP, FGF19 may be decreased in a similar way as to that observed in the AP mouse model.

### Genetic polymorphisms in *FXR* are not associated with acute pancreatitis

To study a potential association between AP and *FXR*, 387 patients with AP and 853 controls were genotyped for 9 tagging and 2 functional SNPs in the FXR locus. An association with AP was seen for one of the variants (rs10860603, p = 0.0364, OR 1.30, 95% CI 1.02–1.65) (Table 1). This association did not, however, withstand correction for the number of tested SNPs (p_corrected_  = 0.40). There was no association of haplotypes of *FXR* with AP (data not shown).

To investigate a potential association between *FXR* and the course and outcome of AP, we studied the prevalence of the genetic variants in patients with a severe course versus a mild course of AP, patients with infected pancreatic necrosis versus patients without it, and patients who died from the pancreatitis versus those who survived (Table S3 in File S1). One of the tag SNPs seemed to be associated with a severe course of AP (rs10860603, p = 0.0368, OR 1.61, 95% CI 1.00–2.60) and one with infection of pancreatic necrosis (rs11110395, p = 0.0099, OR 2.55, 95% CI 1.28–5.07). Haplotypes containing the same risk allele also seemed to show association (data not shown). Another tag SNP showed a significant difference between patients who died and those who survived (rs11837065, p = 0.0272, OR 2.09, 95% CI 1.07–4.06). After Bonferroni correction for multiple testing, however, there were no associations of SNPs or haplotypes in the *FXR* gene with course or outcome of AP. Finally, we compared patients with biliary AP to patients with AP of non-biliary origin. None of the SNPs was associated with a biliary cause of AP (Table S3 in File S1).

## Discussion

Failure of the intestinal barrier plays an important role in human acute pancreatitis, because it facilitates bacterial translocation which can lead to infectious complications. Such complications strongly increase mortality due to pancreatitis. Which specific molecular events eventually lead to failure of the intestinal barrier in acute pancreatitis is still largely unclear. In mouse [31] and rat [32], [33] experimental acute pancreatitis, however, it has been shown that tight junction failure in the pancreas is a very early event. In rats, more specifically, disruption of the actin cytoskeleton and of tight junctions occurring in experimental pancreatitis leads to increased paracellular permeability [32], [34].

Because of the role of FXR in intestinal barrier function, namely prevention of bacterial translocation and modulation of inflammation, we hypothesized that FXR might play an important role in AP. Deficiency of FXR might lead to increased severity of pancreatitis, increased bacterial translocation and subsequent infectious complications. In this study, we therefore explored, with the aid of complementary animal and human experiments, whether FXR could affect AP. We observed that induction of AP by repeated administration of a supraphysiological dose of the CCK-analogue cerulein was accompanied by decreased expression of Fxr target genes in the ileum. However, the ileal Fxr pathway appears to have no major pathogenic role in this model of AP, as indicated by similar pancreatic histopathology following induction of AP in mice with global deficiency of *Fxr* and in wild-type controls. Moreover, a case-control association study indicated that genetic variation in the *FXR* locus is not associated with the risk, etiology or outcome of AP in human subjects. The collective findings of our study indicate that FXR is not a major player in the pathogenesis of AP.

In an initial experiment in wild-type mice, we observed that expression of ileal Fxr target genes *Fgf15*, *Shp* and *Ibabp* (Figure 1C) was disturbed at 24 hrs after induction of AP, while expression levels recovered at the time point that histopathological damage of the pancreas had largely resolved (i.e. 72 hrs after AP induction). Of note, decline of Fxr activity was shown through decreased expression of Fxr target genes, without change of FXR expression. This phenomenon is in line with previous data obtained in patients with Crohn's disease, in animal colitis models, and *in vitro* and *ex vivo* models, where FXR expression itself was not significantly changed by pro-inflammatory cytokines. These findings indicate that the inhibition of FXR target gene expression is due to decreased FXR activity [35]. Decline in ileal FXR target gene expression is likely to be due to impaired delivery of its activating bile salt ligands. It is well known that under pro-inflammatory conditions such as AP, small intestinal motility is decreased, both under fasting and fed conditions, with decreased ileal bile salt delivery as a result [36]. Since AP did not affect bilirubin levels in our wild-type and Fxr^-/-^ mice, post-hepatic obstruction by the inflamed pancreas is unlikely. The transient decline in ileal *Fgf15* expression likely accounted for de-repression of hepatic *Cyp7a1*, as higher expression of this bile salt synthetic gene was found after 24 hrs (data not shown). In our Ussing chamber experiments in wild-type mice, impaired ileal Fxr activation was accompanied by decreased transepithelial resistance at the early time point (Figure 1B), indicative for increased intestinal permeability, without ileal inflammation. In contrast, in our second series of experiments, plasma levels of FITC-conjugated dextran were not increased by AP induction in either wild-type or Fxr^-/-^ mice. These findings indicate that transepithelial resistance measurements are a more sensitive marker for disturbed intestinal permeability than FITC-conjugated dextran. Nevertheless, Fxr^-/-^ mice had significantly higher plasma levels of FITC-conjugated dextran than wild-type mice, which was in line with the increased intestinal permeability in Fxr^-/-^ mice previously reported [13].

Although transient Fxr dysfunction was apparent in the early phase of acute murine pancreatitis, this likely did not have a pathogenic contribution, as the severity of AP was similar in mice with genetic disruption of *Fxr* and controls (Figure 2A and B). The lack of effect upon loss-of-function may relate to decreased intestinal transport of activating bile salt ligands to Fxr in the ileum, as discussed above, and other Fxr expressing tissues of wild-type mice, and result in a phenotype resembling that of the true Fxr-deficient mouse. If this interpretation is correct, gain-of-function studies (e.g. Fxr agonism) may, in theory, be more suitable to address the role of Fxr in AP. Likewise, other models of AP that do not rely on overstimulation of the gallbladder and exocrine pancreas function could, in theory, be employed to further delineate a role of Fxr in AP. Nevertheless, our combined human and murine data strongly argue against a critical role of Fxr in human AP.

In line with our data in wild-type mice, our analysis of non-fasted serum FGF19 levels suggests that ileal FXR dysfunction could also occur in patients with AP. Being a bile salt-regulated enterokine, circulating FGF19 levels increase postprandially in healthy controls (Figure 3). FGF19 levels in enterally fed patients with AP, however, are close to values observed in fasted controls. A possible role of FXR in human AP was further addressed by studying genetic variation at the FXR locus in a cohort of 387 cases and 853 controls. None of the 11 SNPs tested (9 tagging and 2 functional variants), nor the inferred haplotypes, were independently associated with AP. Likewise, none of the variants were independently associated with the risk or course of AP, nor in haplotypes. Thus, genetic variation in the *FXR* locus does not predispose to, or have a major impact on the course of AP in human subjects.

Of note, genetic variation in the FXR locus did not differ between subjects with a biliary (i.e. gallstone) or non-biliary (mainly alcohol) cause of AP. *Fxr^-/-^* mice on a lithogenic diet are highly susceptible to cholesterol gallstone formation due to altered biliary lipid composition [37], although data on the role of FXR in human cholesterol gallstone formation are extremely limited. In female, non-obese gallstone patients, decreased expression of *FXR* and its target genes *ASBT*, ileal lipid binding protein (*ILBP*) and *OSTα-OSTβ* (all involved in bile acid transport) has been described in the enterocyte [38], [39]. These findings suggest an intestinal defect with decreased absorption and subsequently a diminished bile acid pool [40]. Data on *FXR* gene polymorphisms in biliary disease show conflicting results. In a Mexican population, the most commonly found *FXR* haplotype was associated with gallstone prevalence in males, whereas no association was found in German and Chilean populations [41]. Our data yielding an absence of association of *FXR* polymorphisms or haplotypes contribute to knowledge in the subgroup of patients with gallstone pancreatitis. This subgroup is noteworthy for the presence of small gallstones and biliary sludge [42].

In conclusion, loss-of-function of *Fxr* did not affect the severity of pancreatitis in the relatively mild model of cerulein-induced AP (fast recovery, no infection, mild histopathological abnormalities). Moreover, our genetic study does not support a major role for variation in the *FXR* locus as a determinant of human AP.

## Supporting Information

File S1 **Supporting tables. Table S1.** Primer sequences. **Table S2.** SNP information. **Table S3.** Association analysis of genetic variants in *FXR* with subgroups of acute pancreatitis patients.
(DOC)
