# Supplementary material for: Impact of Global Fxr Deficiency on Experimental Acute Pancreatitis and Genetic Variation in the FXR Locus in Human Acute Pancreatitis
Source: PLoS One. 2014 Dec 3;9(12):e114393. doi: 10.1371/journal.pone.0114393 (PMC4255038; doi:10.1371/journal.pone.0114393)
Supplement: File S1 — Supporting tables. Table S1. Primer sequences. Table S2. SNP information. Table S3. Association analysis of genetic variants in FXR with subgroups of acute pancreatitis patients. (DOC) [file pone.0114393.s001.doc]

**Supplementary Table 1.** Primer sequences

| **Gene** | **Forward primer** | **Reverse primer** |
| --- | --- | --- |
| FXR | 5'tgagaacccacagcatttcg3' | 5'gcgtggtgatggttgaatgtc3' |
| SHP | 5'cgatcctcttcaacccagatg3' | 5'agggctccaagacttcacaca3' |
| FGF15 | 5’aaaacgaacgaaatttgttggaa3’ | 5`acgtccttgatggcaatcg3` |
| IBABP | 5’ttgagagtgagaagaattacgatgagt3’ | 5'tttcaatcacgtctccctggaa3' |
| ASBT | 5'tgactcgggaacgattgtg3' | 5'ggaataacaagagcaaccagagaa3' |
| iNOS | 5'caggaggagagagatccgattta3' | 5'gcattagcatggaagcaaaga3' |
| ANG1 | 5’agcgaatggaagcccttaca3’ | 5’ctcatcgaagtggaccggca3’ |
| IL18 | 5'ccgcctcaaaccttcca3' | 5'catggcagccattgttcct3' |
| CAR12 | 5’ctcagacctgtaccctgacttca3’ | 5’gagcctatctcaataagaacagcaa3’ |
| TNF-α | 5’catcttctcaaaattcgagtgacaa3’ | 5’tgggagtagacaaggtacaaccc3’ |
| Cyclophilin | 5'ggagatggcacaggaggaa3' | 5'gcccgtagtgcttcagctt3' |

**Supplementary Table 2.** SNP information

|  | **SNP number** | **Chromosomal location (12)** |
| --- | --- | --- |
| **Tagging SNPs** | rs11837065 | 100859983 |
|  | rs12313471 | 100864393 |
|  | rs11110390 | 100874901 |
|  | rs4764980 | 100885107 |
|  | rs11110395 | 100888664 |
|  | rs17030285 | 100930213 |
|  | rs11610264 | 100932375 |
|  | rs10860603 | 100943948 |
|  | rs35739 | 100948515 |
| **Functional SNPs** | -1g>t, rs56163822 | exon 3 |
|  | 518t>c, rs61755050 | exon 5 |

**Supplementary Table 3.** Association analysis of genetic variants in *FXR* with subgroups of acute pancreatitis patients

|  | **Patients with severe vs. patients with mild AP** | **Patients with infected pancreatic necrosis vs. patients without it** | **Patients who died vs. surviving patients** | **Patients with biliary vs. non-biliary acute pancreatitis** |
| --- | --- | --- | --- | --- |
|  | OR (95% CI) | OR (95% CI) | OR (95% CI) | OR (95% CI) |
| -1 G>T | 1.63 (0.42-6.42) | 1.18 (0.22-6.46) | 2.62 (0.47-14.59) | 1.47 (0.51-4.24) |
| 518 T>C | 2.28 (0.13-41.40) | 1.01 (0.06-18.33) | 0.33 (0.02-6.10) | 1.54 (0.30-7.84) |
| rs11837065 | 1.35 (0.96-1.90) | 1.18 (0.76-1.83) | 2.09 (1.07-4.06)^#^ | 1.00 (0.73-1.37) |
| rs12313471 | 1.02 (0.50-2.11) | 1.61 (0.53-4.92) | 0.71 (0.19-2.65) | 1.02 (0.54-1.94) |
| rs11110390 | 1.24 (0.88-1.73) | 1.27 (0.83-1.94) | 1.69 (0.88-3.24) | 1.07 (0.79-1.45) |
| rs4764980 | 1.17 (0.85-1.62) | 1.35 (0.89-2.04) | 1.57 (0.81-3.06) | 1.09 (0.82-1.46) |
| rs11110395 | 1.64 (0.87-3.07) | 2.55 (1.28-5.07)^*^ | 1.20 (0.23-6.35) | 1.41 (0.76-2.62) |
| rs17030285 | 1.35 (0.82-2.20) | 1.01 (0.55-1.84) | 1.20 (0.44-3.28) | 1.17 (0.77-1.77) |
| rs11610264 | 1.03 (0.72-1.47) | 1.22 (0.76-1.96) | 0.99 (0.48-2.05) | 1.00 (0.72-1.39) |
| rs10860603 | 1.61 (1.00-2.60)^ | 1.18 (0.66-2.10) | 1.14 (0.45-2.88) | 1.31 (0.88-1.94) |
| rs35739 | 1.16 (0.84-1.60) | 1.11 (0.74-1.67) | 1.20 (0.63-2.31) | 1.18 (0.89-1.58) |

OR = odds ratio; 95% CI = 95% confidence interval

^rs10860603, 88.1% vs. 81.7%, p = 0.0368; ^*^rs11110395, 11.5% vs. 5.1%, p = 0.0099; ^#^rs11837065, 52.8% vs. 34.7%, p = 0.0272.
